# Genetic variants in RNA m^5^C modification genes associated with survival and chemotherapy efficacy of colorectal cancer

**DOI:** 10.1002/cam4.5018

**Published:** 2022-07-21

**Authors:** Silu Chen, Xiangming Cao, Shuai Ben, Lingjun Zhu, Dongying Gu, Yuan Wu, Shuwei Li, Qiang Yu

**Affiliations:** ^1^ Department of Gastroenterology The Affiliated Suzhou Hospital of Nanjing Medical University, Suzhou Municipal Hospital, Gusu School, Nanjing Medical University Jiangsu China; ^2^ Department of Genetic Toxicology, The Key Laboratory of Modern Toxicology of Ministry of Education, Center for Global Health, School of Public Health Nanjing Medical University Nanjing China; ^3^ Department of Environmental Genomics, Jiangsu Key Laboratory of Cancer Biomarkers, Prevention and Treatment, Collaborative Innovation Center for Cancer Personalized Medicine Nanjing Medical University Nanjing China; ^4^ Department of Oncology The Affiliated Jiangyin Hospital of Southeast University Medical College Jiangyin China; ^5^ Department of Oncology The First Affiliated Hospital of Nanjing Medical University Nanjing China; ^6^ Department of Oncology Nanjing First Hospital, Nanjing Medical University Nanjing China; ^7^ Department of Medical Oncology Jiangsu Cancer Hospital, Jiangsu Institute of Cancer Research, The Affiliated Cancer Hospital of Nanjing Medical University Nanjing China

**Keywords:** chemotherapy, colorectal cancer, genetic variants, survival

## Abstract

**Background:**

Colorectal cancer is one of the most common malignant digestive tract tumors with a poor prognosis. RNA 5‐methylcytosine (m^5^C) is an important posttranscriptional widespread modification involved in many biological processes. However, the association between genetic variations of m^5^C modification genes and the prognostic value of colorectal cancer remains unclear.

**Methods:**

We investigated the association between candidate single nucleotide polymorphisms (SNPs) in 13 m^5^C modification genes and colorectal cancer overall survival (OS) after chemotherapy by the Cox regression model. The combined effect of selected SNPs on OS, progression‐free survival (PFS), and disease control rate (DCR) was assessed by the number of risk alleles (NRA). The GTEx and TCGA database were used to perform expression qualitative trait locus (eQTL) analysis.

**Results:**

We identified that two SNPs in *YBX1* were associated with OS after chemotherapy (HR = 1.43, *p* = 0.001 for rs10890208; HR = 1.36, *p* = 0.025 for rs3862218). A striking dose–response effect between NRA and OS after chemotherapy was found (*p*_trend_ = 0.002). The DCR of patients receiving oxaliplatin chemotherapy in the 3–4 NRA group was markedly reduced in comparison to that in the 0–2 NRA group (OR = 1.49, *p* = 0.036). Moreover, *YBX1* mRNA expression was significantly overexpressed in tumor tissues (*p* < 0.05) in the TCGA database, and eQTL analysis demonstrated that the two SNPs were associated with *YBX1* (*p* = 0.003 for rs10890208 and *p* = 0.024 for rs3862218).

**Conclusion:**

Our study indicates that genetic variants in m^5^C modification genes may mediate changes in *YBX1* mRNA levels and affect the chemotherapeutic efficacy of colorectal cancer patients.

## Graphical abstract

We identified that rs10890208 and rs3862218 in *YBX1* were associated with colorectal cancer overall survival after chemotherapy. Our study indicates that genetic variants in m^5^C modification genes may mediate changes in *YBX1* mRNA levels and affect the chemotherapeutic efficacy of colorectal cancer patients.

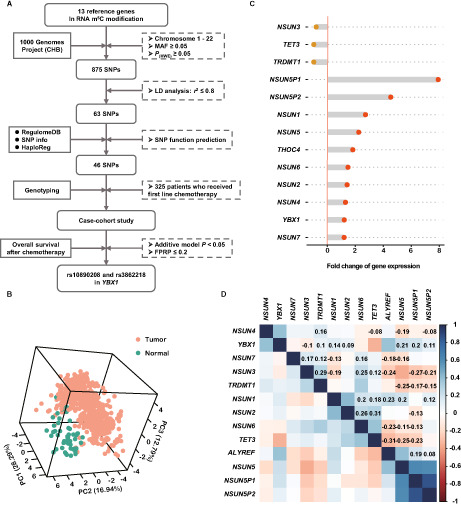

## 1 INTRODUCTION

Colorectal cancer is the fourth leading cause of cancer‐related deaths with almost 900,000 deaths annually in the world.^1^ Due to the lack of specific symptoms in the early stage of colorectal cancer, the disease has progressed to a severe stage with palpable clinical symptoms. In the surgical treatment of colorectal cancer, surgical resection followed by chemotherapy has become the main therapeutic regimen. Chemotherapy is one of the feasible options for most patients with colorectal cancer.^2^ However, as a common severe disease, there are significant differences in clinical efficacy and prognosis of different colorectal cancer patients undergoing chemotherapy. Genomewide association analysis has identified many single nucleotide polymorphisms (SNPs) of colorectal cancer susceptibility and prognosis,^3^, ^4^, ^5^ but there are few studies on the efficacy and drug resistance of chemotherapy in colorectal cancer patients.^6^

RNA methylation is an abundant posttranscriptional modification for most types of RNA molecules. To date, more than 100 different types of RNA modifications have been reported, including N6‐methyladenosine (m^6^A), 7‐methylguanosine (m^7^G), N1‐methyladenosine (m^1^A), 5‐methylcytosine (m^5^C), and pseudouridine (Ψ).^7^ In our previous study, we found that genetic variants in *SND1* in the m^6^A modification contribute to the risk of colorectal cancer.^8^ As a modification of RNA that has gained increasing attention in recent years, methylation of carbon 5 in cytosine was first detected in highly abundant and stable tRNAs and rRNAs.^9^, ^10^ Accumulating evidence has revealed that RNA m^5^C modification may play vital regulatory roles in tumorigenesis and cancer progression.^11^, ^12^, ^13^ For example, the expression of m^5^C “reader” *YBX1* is related to the sorafenib resistance in hepatocellular cancer.^14^ Cheng et al. found that m^5^C modification and m^5^C “writer” *NSUN1* may mediate chromatin organization and 5‐azacytidine response and resistance in leukemia.^15^ However, the specific prognostic value of genetic variants in m^5^C modification genes in cancer patients treated with chemotherapy, including colorectal cancer patients, remains largely unknown.

Previous studies have suggested the important role of RNA m^5^C modification in cancer progression and chemotherapy sensitivity.^16^, ^17^ However, few studies focused on the genetic variant in m^5^C modification genes.^18^ Therefore, to further understand the genetic variations of m^5^C modification pathway genes and its association with the survival time of patients with colorectal cancer after chemotherapy, we conducted a prospective study to clarify the role of RNA m^5^C modification in chemotherapy response to colorectal cancer using a path‐based approach.^19^

## 2 MATERIALS AND METHODS

### 2.1 Study participants

This study included a case‐cohort of 344 colorectal cancer patients from the First Affiliated Hospital of Nanjing Medical University and the Affiliated Nanjing First Hospital, who were recruited in September 2010 and were followed up by telephone interviews. A total of 19 patients who did not receive chemotherapy were excluded. Detailed information has been elucidated in our previous article.^20^

Overall survival (OS) after chemotherapy was deemed the major endpoint in our analysis. It was calculated from the time of first chemotherapy to the time of last follow‐up or death. The time elapsed between the onset of chemotherapy and objective disease progression, death, or last follow‐up was defined as progression‐free survival (PFS). Tumor response was categorized into four groups, including progressive disease (PD), stable disease (SD), partial response (PR), and complete response (CR). The disease control rate (DCR) was defined as CR, PR, and SD proportion. Nanjing Medical University Institutional Review Board has reviewed and approved the study protocol and each participant gave informed consent.

### 2.2 Selection of genes

The key RNA m^5^C modification genes were selected from previously reported studies. Enzymes have been identified as responsible for m^5^C RNA modification including m^5^C methyltransferases, demethylases, and “readers”. Methylation at the C5 position of RNAs is mediated by m^5^C methyltransferases, also called m^5^C “writer”, including the *NOL1*/*NOP2*/sun domain (*NSUN1*), RNA methyltransferase family (*NSUN1*/*NOP2*, *NSUN2*, *NSUN3*, *NSUN4*, *NSUN5*, *NSUN5P1*/*NSUN5B*, *NSUN5P2*/*NSUN5C*, *NSUN6*, and *NSUN7*), and tRNA aspartic acid methyltransferase 1 (*TRDMT1*/*DNMT2*).^16^, ^21^, ^22^, ^23^ Then, methylated mRNAs are recognized and bound by different “reader” proteins, such as Aly/REF export factor (*ALYREF*) and Y‐box binding protein 1 (*YBX1*).^11^, ^24^ The RNA demethylases Tet methylcytosine dioxygenase 3 (*TET3*) remove the m^5^C modification by degrading the written methylation.^25^ A total of 13 autosomal genes involved in RNA m^5^C modification were used as the candidate genes.

### 2.3 Selection and genotyping of SNPs

SNPs within m^5^C modification genes and their ±2 kb flanking regions were screened by the Chinese Han population in Beijing (CHB) population data from the 1000 Genomes Project (March 2012). The quality control for extracting SNPs included Hardy–Weinberg equilibrium (HWE) ≥ 0.05, minor allele frequency (MAF) ≥ 0.05, pairwise linkage disequilibrium (LD) analysis *r* ^2^ ≤ 0.8. Then, we used online tools including HaploReg (http://compbio.mit.edu/HaploReg),^26^ RegulomeDB (http://regulome.stanford.edu/),^27^ and SNPinfo (http://snpinfo.niehs.nih.gov/)^28^ to distinguish potentially representative functional SNPs. For candidate SNPs, we performed the in silico analysis through UCSC (http://genome.ucsc.edu/), FAVOR (http://favor.genohub.org/),^29^ RNAfold (https://rna.tbi.univie.ac.at/cgi‐bin/RNAWebSuite/RNAfold.cgi/),^30^ 3DSNP2 (https://www.omic.tech/3dsnpv2/),^31^ and Open Targets Genetics (https://genetics.opentargets.org).^32^

Our previous study has described the methods used to extract genomic DNA and perform SNP genotyping.^4^ By the Qiagen Blood kit (Qiagen), we extracted genomic DNA from the blood samples of all study participants. All the SNPs were genotyped using Illumina Human Omni ZhongHua Bead Chips. SNPs with call rates lower than 5% were excluded from our study.

### 2.4 Gene expression analysis

We used the mRNA expression data of colorectal cancer from the Gene Expression Omnibus (GEO) datasets and The Cancer Genome Atlas (TCGA) database (http://cancergenome.nih.gov/) to compare significant differences between tumor tissues and adjacent tissues. The TCGA database included 51 normal colorectal tissues and 625 colorectal cancer tumor tissues. Gene expression data were subjected to log10 transformations uniformly. Besides, expression quantitative trait loci (eQTL) analysis was performed based on the TCGA database and the Genotype Tissue Expression (GTEx) project (http://www.gtexportal.org/). In the GTEx database, 246 subjects were transverse colon samples and 203 subjects were sigmoid colon samples, respectively. Furthermore, we applied restricted cubic spline (RCS) models with five knots at the 5th, 27.5th, 50th, 72.5th, and 95th percentiles of *YBX1* mRNA expression to explore the nonlinear association. The protein interaction network of m^5^C genes is constructed using the online tool STRING (https://string‐db.org/).

### 2.5 Statistical analysis

The principal component analysis (PCA) was used to distinguish the tumor and normal groups. We used unconditional univariate and multivariate Cox regression analyses to estimate the associations between SNPs and clinical characteristics and OS or PFS of the patients with colorectal cancer, and hazard ratio (HR) with its 95% confidence interval (CI) was used to estimate the associations. Survival curves were performed by the Kaplan–Meier method. To evaluate the correlation between DCR and genetic variants, we calculated the crude and adjusted odds ratio (OR) and 95% CI by the logistic regression model. Due to the high correlation between SNPs, multiple test correction was performed with false‐positive report probability (FPRP). A prior probability of 0.10 along with an HR of 1.5 was assigned for FPRP calculation. An FPRP value ≤ 0.20 were defined as a statistically significant association.^33^ We assessed the combined effect of selected SNPs on survival time by the sum of the number of risk alleles (NRA). Significant differences in mRNA expression were compared by Student's *t‐*test or a two‐sided Mann–Whitney *U* test, depending on the variable's type. A linear regression model was used to calculate the association among candidate SNPs and gene expression of their location. We use RNAm^5^Cfinder (http://www.rnanut.net/rnam5cfinder) to predict m^5^C modification sites in RNA sequences. LD analysis was performed by the HaploView 4.2 software. Primary statistical analysis was performed by R software (version 3.5.3). PLINK 1.09 was conducted for other statistical analyses unless otherwise specified. *p* values <0.05 were considered statistically significant.

## 3 RESULTS

### 3.1 Gene and SNP selection

**FIGURE 1:**
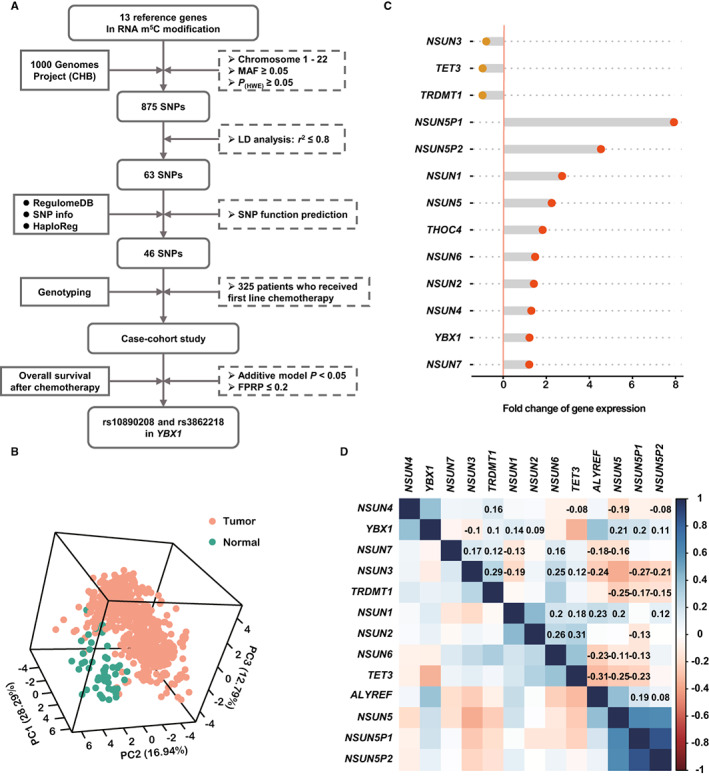
The procedures of selecting colorectal cancer functional genetic variants. Schematic flow for selecting SNPs in 13 genes in RNA m^5^C modification (A). Principal component analysis (PCA) (B), fold change (C), and correlation plot (D) of the 13 genes in the RNA m^5^C modification pathway. CHB, Chinese Han population in Beijing; FPRP, false‐positive report probability; HWE, Hardy–Weinberg Equilibrium; LD, linkage disequilibrium; MAF, minor allele frequency; SNP, single nucleotide polymorphism.

The specific interaction of the m^5^C “writers”, “readers”, and “erasers” has been demonstrated in Figure S1A. Additionally, previous studies have identified the mechanism of these m^5^C modification genes (Table S1). The protein interaction network of these genes is shown in Figure S1B. The schematic flow for selecting RNA m^5^C modification genes and SNPs is shown in Figure 1A. The fold changes of 13 genes are shown in Figure 1B. Principal component analysis shows that 13 genes greatly distinguished the tumor from the adjacent tissue (Figure 1C). The correlation of the candidate genes was demonstrated in Figure 1D. After LD analysis, 63 SNPs remained for further analysis (Table S2). Altogether, 46 SNPs were retained for genotyping analyses after SNP function prediction.

### 3.2 Association between SNPs in RNA m^5^C modification and colorectal cancer prognosis

The association between the candidate SNPs and OS of the patients who receive chemotherapy was shown in Table S3. Four SNPs (rs10890208, rs3862218, and rs12030724 in *YBX1.* and rs11254419 in *TRDMT1*) were markedly associated with OS in an additive model of colorectal cancer patients after adjustment (HR = 1.43, 95% CI = 1.15–1.78, *p* = 0.001; HR = 1.36, 95% CI = 1.04–1.78, *p* = 0.025; HR = 0.73, 95% CI = 0.56–0.97, *p* = 0.028; HR = 1.30, 95% CI = 1.00–1.69, *p* = 0.047, respectively). When a prior probability of 0.1 was assigned, FPRP of 0.018 and 0.184 was observed for *YBX1* rs10890208 and rs3862218, respectively. Consequently, we focused on these two SNPs with moderate linkage (*r* ^2^ = 0.32) in *YBX1* for subsequent analysis.

**FIGURE 2:**
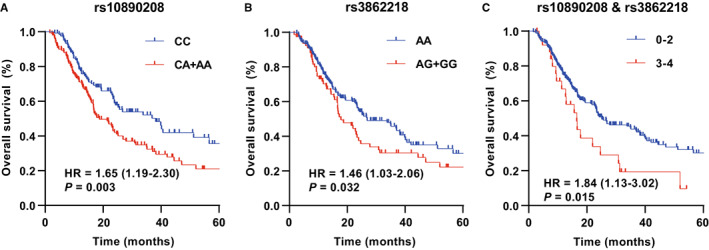
Association between candidate SNPs and *YBX1* in RNA m^5^C modification and overall survival after chemotherapy. Kaplan–Meier curve for rs10890208 (A), rs3862218 (B), and combined alleles (C) based on followed‐up patients.

We assessed the effects of the two candidate SNPs on OS after adjusting the covariates in multivariable Cox regression analyses using four genetic models (additive, codominant, recessive, and dominant; Table 1). In addition to the additive model, rs10890208 in *YBX1* was also associated with reduced OS the patients in the codominant, dominant, and recessive models (HR = 1.55, 95% CI = 1.08–2.21, *p* = 0.017; HR = 1.98, 95% CI = 1.24–3.14, *p* = 0.004; HR = 1.65, 95% CI = 1.19–2.30, *p* = 0.003; HR = 1.62, 95% CI = 1.06–2.48, *p* = 0.026, respectively) after adjustment. Besides, significant association was found between rs3862218 and reduced OS of the patients in the dominant models (HR = 1.46, 95% CI = 1.03–2.06, *p* = 0.032). The association of colorectal cancer and risk genotypes of *YBX1* rs10890208 and rs3862218 was illustrated by Kaplan–Meier curves (Figure 2A,B). Then, we evaluated the risk effect between the two SNPs and OS in colorectal cancer patients by stratified analysis. Table S4 shows that rs10890208 C > A significantly reduced the overall survival of irinotecan patients (HR = 1.75, 95% CI = 1.03–2.96, *p* = 0.038). On the contrary, the risk effect of rs3862218 A > G was more significant in the subgroups of oxaliplatin patients (HR = 1.72, 95% CI = 1.06–2.80, *p* = 0.029).

**TABLE 1:** Association of rs10890208 and rs3862218 with OS after chemotherapy of colorectal cancer in four genetic models

Models	Death/All	HR (95% CI)	*p*	HR (95% CI)	*p* ^a^
rs10890208					
CC	58/122	1.00		1.00	
CA	65/111	1.48 (1.04–2.11)	0.031	1.55 (1.08–2.21)	0.017
AA	27/40	1.95 (1.23–3.09)	0.004	1.98 (1.24–3.14)	0.004
Additive model		1.41 (1.13–1.76)	0.002	1.43 (1.15–1.78)	0.001
Dominant model		1.59 (1.14–2.21)	0.006	1.65 (1.19–2.30)	0.003
Recessive model		1.61 (1.06–2.45)	0.025	1.62 (1.06–2.48)	0.026
rs3862218					
AA	100/195	1.00		1.00	
AG	41/67	1.32 (0.92–1.90)	0.137	1.41 (0.97–2.04)	0.069
GG	9/11	1.66 (0.84–3.28)	0.148	1.75 (0.88–3.50)	0.111
Additive model		1.30 (1.00–1.70)	0.053	1.36 (1.04–1.78)	0.025
Dominant model		1.37 (0.97–1.92)	0.071	1.46 (1.03–2.06)	0.032
Recessive model		1.54 (0.78–3.02)	0.211	1.60 (0.81–3.15)	0.179
Number of risk allele (NRA)^b^					
0	58/122	1.00		1.00	
1	34/59	1.56 (1.02–2.39)	0.039	1.60 (1.04–2.47)	0.032
2	39/66	1.43 (0.95–2.15)	0.084	1.49 (0.99–2.25)	0.054
3	10/15	2.39 (1.21–4.70)	0.012	2.53 (1.27–5.01)	0.008
4	9/11	1.95 (0.97–3.95)	0.062	2.07 (1.02–4.22)	0.044
Trend test			0.004		0.002
0–2	131/247	1.00		1.00	
3–4	19/26	1.76 (1.08–2.85)	0.022	1.84 (1.13–3.02)	0.015

Abbreviations: CI, confidence interval; HR, hazard ratio; NRA, number of the risk allele; OS, overall survival.

^a^ Adjusted for age, sex, smoking, and drinking status in Cox regression models.

^b^ NRA for OS included rs10890208 A and rs3862218 G alleles. Some cases were not included due to incomplete overall survival information or genotype.

We further analyze the associations between these two SNPs in *YBX1* and PFS or DCR by four genetic models (additive, codominant, recessive, and dominant), but we found no significant associations between them and PFS or DCR of colorectal cancer (Table S5).

### 3.3 Survival of colorectal cancer patients with combined risk alleles

To assess the combined association between the two candidate SNPs and colorectal cancer survival, we added the number of *YBX1* rs10890208 A allele and rs3862218 G allele as an NRA score. First, we categorized five groups for all the patients based on the number of risk alleles (i.e., 0, 1, 2, 3, and 4). Furthermore, the trend test indicated a significant risk dose–response effect (*p*_trend_ = 0.002) in OS (Table 1). Then we assorted all patients into 0–2 NRA groups and 3–4 NRA groups to observe the prognosis of people with more allelic mutations. Compared with 0–2 NRA group, 3–4 NRA group had a significantly lower colorectal cancer OS (HR = 1.84, 95% CI = 1.13–3.02, *p* = 0.015). Kaplan–Meier curves showed OS after chemotherapy for the two NRA groups of colorectal cancer (Figure 2C). However, Table S5 shows that the dose–response effect of NRA was not significant in PFS and DCR.

### 3.4 Effect of combined risk alleles on colorectal cancer by stratified analyses

To further investigate the combined effect of risk alleles, stratified analyses were performed to assess the association between the different NRA groups and OS of colorectal cancer patients (Table 2). For lifestyle‐related characteristics, compared with the 0–2 NRA group, patients in the 3–4 NRA group showed significantly poorer survival, which is evident in females (HR = 3.58, 95% CI = 1.61–7.99, *p* = 0.002). Besides, significant multiplicative interaction was found between the two SNPs and sex (*p* = 0.026). In terms of clinicopathological features, patients who suffered from rectal cancer in the 3–4 NRA group had a markedly poorer survival (HR = 3.27, 95% CI = 1.63–6.58, *p* = 0.001). Well and moderately differentiated colorectal cancers (HR = 1.91, 95% CI = 1.04–3.52, *p* = 0.037) had an increased risk of survival. Dukes stage D (HR = 1.86, 95% CI = 1.12–3.09, *p* = 0.016) and organ number of metastases less than two (HR = 2.04, 95% CI = 1.16–3.60, *p* = 0.014) were also found a significant higher colorectal cancer death risk. However, no significant death risk was observed between the oxaliplatin‐based treatment (*p* = 0.094) and irinotecan‐based treatment (*p* = 0.100) subgroups.

**TABLE 2:** Stratified analysis of the risk alleles^a^ of rs10890208 and rs3862218 on OS after chemotherapy

Variables	HR (95% CI)^b^	*p* ^b^	*p*_interaction_
Age (years)			
≤60	1.75 (0.86–3.53)	0.121	0.300
>60	1.84 (0.92–3.66)	0.082	
Sex			
Female	3.58 (1.61–7.99)	0.002	0.026
Male	1.34 (0.70–2.54)	0.375	
Smoking status			
Never	1.58 (0.71–3.50)	0.262	0.566
Ever	2.01 (1.04–3.89)	0.039	
Drinking status			
Never	1.74 (0.78–3.86)	0.177	0.566
Ever	2.12 (1.10–4.10)	0.025	
Tumor site			
Colon	1.09 (0.49–2.42)	0.841	
Rectum	3.27 (1.63–6.58)	0.001	
Tumor grade			
Well + Moderate	1.91 (1.04–3.52)	0.037	
Poor	1.94 (0.77–4.85)	0.159	
Dukes stage			
C	0.17 (0.00–11.59)	0.413	
D	1.86 (1.12–3.09)	0.016	
Metastasis^c^			
≤2	2.04 (1.16–3.60)	0.014	
>2	1.59 (0.51–4.91)	0.422	
Treatment			
Oxaliplatin	1.92 (0.90–4.10)	0.094	
Irinotecan	1.77 (0.90–3.48)	0.100	

Abbreviations: CI, confidence interval; HR, hazard ratio; OS, overall survival.

^a^ Risk alleles included rs10890208 A and rs3862218 G alleles.

^b^ Adjusted for age, sex, smoking, and drinking status in Cox regression models; some cases were not included due to incomplete overall survival information.

^c^ Some cases were not included due to missing clinical data.

**FIGURE 3:**
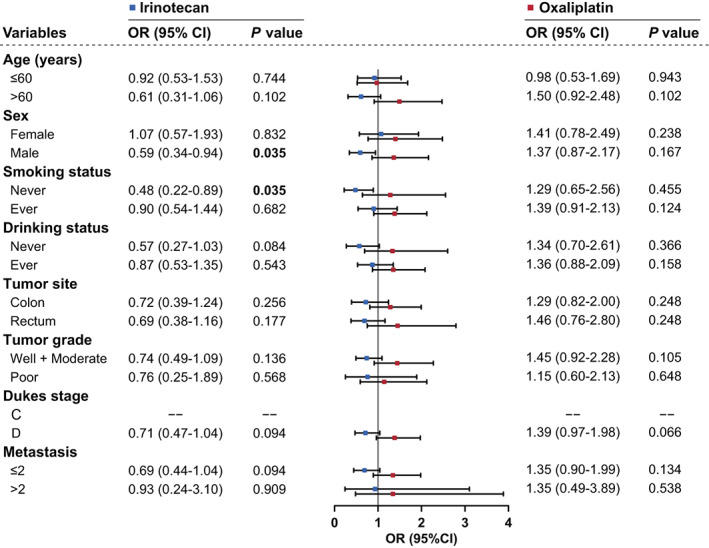
Stratification analyses of the associations between NRA and colorectal cancer DCR. Each point and horizontal line represent OR and 95% CI.

In addition, stratified analyses were also conducted to assess the association between NRA, PFS, and DCR of colorectal cancer (Table S6). The result showed that the disease control rate of patients receiving oxaliplatin chemotherapy in the 3–4 NRA group was significantly reduced in comparison with that in the 0–2 NRA group (OR = 1.49, 95% CI = 1.02–2.19, *p* = 0.036). To further explore the sensitivity to different chemotherapy regimens of the different NRA groups, we performed stratified analyses of DCR in both chemotherapy regimens. In general, oxaliplatin reduced DCR in 3–4 NRA group patients, with significant effects in older or high malignancy patients (Figure 3). On the contrary, irinotecan increased DCR in 3–4 NRA group patients, especially for male and nonsmoker (OR = 0.59, 95% CI = 0.34–0.94, *p* = 0.035; OR = 0.48, 95% CI = 0.22–0.89, *p* = 0.035, respectively).

### 3.5 Functional annotation and eQTL analysis

To further explore the function of the SNPs, we predicted the function of the two SNPs and their strong LD SNPs based on the online prediction tools (Table S7). The position of rs10890208 and rs3862218 and the predicted m^5^C sites in *YBX1* are shown in Figure S2A,B. In addition, we found the histone marks (H3K27ac, H3K36me3, H3K4me1, and H3K4me3) in the transverse colon (Figure S2C) and sigmoid colon tissue (Figure S2D) of the two SNPs via the ChIP‐Seq data in UCSC. An integrative score calculated by FAVOR showed strong functional signals of epigenetics, local nucleotide diversity, and transcription factor binding (Figure S2E). RNAfold predicted the change of minimum free energy (MFE) caused by two SNP alleles. ΔMFE is calculated by subtracting the wild‐type MFE from the mutant MFE (ΔMFE = −1.9 kcal/mol for both rs10890208 and rs3862218, Figure S3). Additionally, 3DSNP2 revealed transcription factor binding site (TFBS) of the two SNPs (*CTCF* for rs10890208; *POLR2A*, *SMARCA4*, *TRIM28* for rs3862218). We further analyzed the correlation between this TFBS and *YBX1* and the different genotypes of the two SNPs. Our prediction analysis illustrated that the risk genotype rs10890208 AA could alter the binding ability to *CTCF* (Figure S4A). Analogously, the risk genotype rs3862218 GG could alter the binding ability to *POLR2A* but not *SMARCA4* and *TRIM28* (Figure S4B–D). Finally, we applied the PheWAS strategy to detect the two SNPs and phenome via the Open Targets Genetics Portal. As shown in Figure S5, rs10890208 and rs3862218 were both associated with gastrointestinal disease.

**FIGURE 4:**
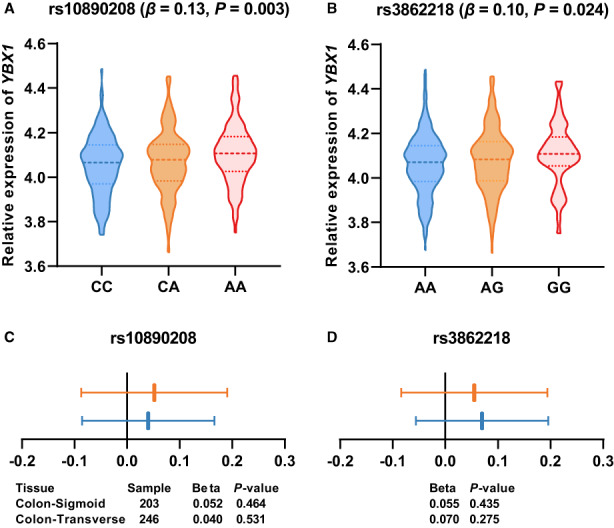
The eQTL between rs10890208 and rs3862218 and *YBX1* in colorectal cancer. Data from TCGA (A, B) and GTEx (colon‐transverse and colon‐sigmoid cancers) (C, D). Effect size (beta): the slope of the linear regression of normalized expression data versus the three genotype categories using single‐tissue eQTL analysis.

Then, we conducted an eQTL analysis to assess the correlation between these two SNPs and the mRNA expression of *YBX1*. In the TCGA database, we found that the *YBX1* expression level increased with the NRA of rs10890208 and rs3862218 in colorectal cancer (*p* = 0.003 and 0.024, respectively). However, no significant association was observed among *YBX1* and rs10890208 (*p* = 0.464 for sigmoid; *p* = 0.531 for transverse) and rs3862218 (*p* = 0.435 for sigmoid; *p* = 0.275 for transverse) in the GTEx database (Figure 4).

### 3.6 Aberrant expression of *YBX1* in colorectal cancer tissues

**FIGURE 5:**
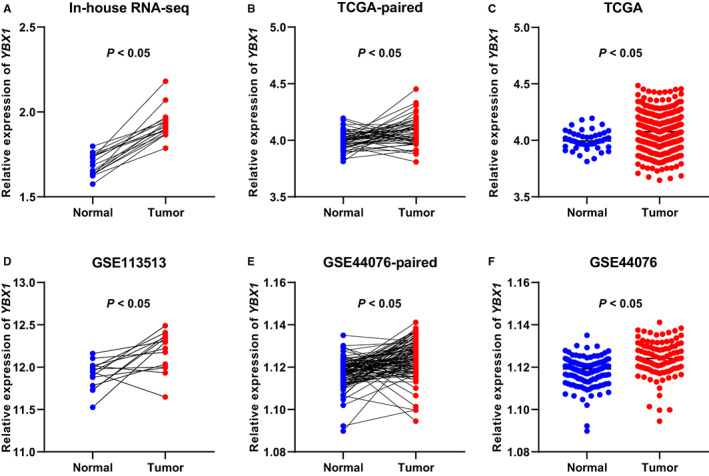
*YBX1* had significantly higher expression levels in colorectal cancer tumor tissues than in normal tissues. *YBX1* mRNA expression levels in 17 paired clinical samples (A), TCGA database (B, C), and the GEO database (GSE113513 and GSE44076) (D–F).

To investigate the function mechanism of *YBX1*, we evaluated the mRNA expression level of *YBX1*. It was significantly increased in tumors than in normal samples in in‐house RNA‐Seq data, TCGA paired data, TCGA database, GSE113513, GSE44076 paired data, and GSE44076 (*p* < 0.05) in Figure 5. Moreover, in the TCGA database, we compared the OS between colorectal cancer with high expression levels of *YBX1* and those with low levels of expression. The correlation was not statistically significant between *YBX1* expression levels and OS in patients suffering from both colon and rectal cancer (*p* = 0.190, 0.930, respectively; Figure S6A). However, the increased expression of *YBX1* was associated with better overall survival in adrenocortical carcinoma, liver hepatocellular carcinoma, mesothelioma, and sarcoma patients (*p* < 0.05).

To further explore whether *YBX1* is associated with colorectal cancer development, we analyzed the relationship between *YBX1* and the colorectal cancer stage. However, *YBX1* expression was not statistically significant with tumor stage (tumor stage III and IV vs tumor stage I and II; OR = 0.61, *p* = 0.406; OR_adj_ = 0.64, *p* = 0.465; Table S8), although it was significantly compared with normal tissues (Figure S6B). Interestingly, we found a dramatic dose–response effect of *YBX1* mRNA expression on the tumor stage (*p*_nonlinear_ <0.05; Figure S6C). It suggested that patients with extremely high or extremely low *YBX1* expression are more likely to suffer from higher colorectal cancer progression risk.

## 4 DISCUSSION

For cancer patients who receive chemotherapy, sensitivities to chemotherapeutic drugs in individuals differ greatly. Therefore, a great deal of research has focused on the identification of novel biomarkers, thus offering a scenario of individualized cancer therapy.^34^ A recent study shows that the role of m^5^C‐related regulators in hepatocellular carcinoma is dysregulated and associated with patient survival.^35^ Several reports indicate that m^5^C modification genes are associated with chemotherapy resistance.^36^, ^37^ However, there is still a lack of research on the genetic variations in m^5^C pathway genes and clinical outcomes of colorectal cancer patients. We investigated the association between genetic variations of 13 m^5^C modification genes and the OS of patients with colorectal cancer after chemotherapy through a cohort follow‐up data set. We found that two SNPs in *YBX1* were independently related to the survival of colorectal cancer patients. Therefore, the *YBX1* rs10890208 and rs3862218 may predict a reduction in the overall survival of colorectal cancer patients treated with chemotherapy.

The SNP rs10890208 and rs3862218 are located in *YBX1*, Y‐box‐binding protein 1, which can encode proteins that bind both DNA and RNA and thus participate in numerous cellular processes including pre‐mRNA splicing, mRNA packaging, DNA reparation, and mediating oncogenic transcription and translation.^38^, ^39^, ^40^ Aberrant expression of *YBX1* is associated with uncontrolled cellular proliferation of cancer. For example, the suppression of *YBX1* in the HT‐29, HCT‐116, and CaCo2 cell lines decreased cell proliferation and migration.^41^ In recent years, research demonstrated new evidence that *YBX1* may have a role in microRNA processing and contribute to the invasive characteristics of Pleural Mesothelioma.^42^ As an m^5^C “reader”, *YBX1* recruits *ELAVL1* to maintain the stability of its target mRNA.^11^ Interestingly enough, *YBX1* has been reported as a prognostic marker for poor outcome and chemotherapy efficacy in colorectal cancer, breast cancer, nasopharyngeal carcinoma, and many other cancers.^43^, ^44^, ^45^ Moreover, *YBX1* could induce oxaliplatin resistance in colon adenocarcinoma cell lines by induction of NONO and RALY proteins.^46^ Our findings revealed a higher *YBX1* mRNA expression in colorectal tumor tissues than in adjacent tissues. These studies have shown that *YBX1* is closely related to many kinds of cancer occurrence and development, but further mechanism studies are therefore warranted to validate the function of *YBX1*.

We further estimated the association of overall survival in colorectal cancer patients receiving chemotherapy with rs10890208 and rs3862218 in *YBX1*. The two SNPs have significantly combined effects on reducing the OS of patients and such effects occur in a dose‐dependent manner. We thus performed a stratified analysis to ascertain whether this combined effect on survival and chemotherapy response existed in environmental or clinical factors. This study implied that females undergoing chemotherapy had a significant risk of death, which was consistent with the funding that women treated with adjuvant fluorouracil chemotherapy had an increased risk of poisoning in a cohort study.^47^ Furthermore, rs10890208 and rs3862218 were significantly associated with OS in the subgroup with rectum cancer and Dukes stage D. These results suggest that treatment with genotypes may have a positive effect on overall survival when tumors occur in the rectum or are more severe.

For the two chemotherapy regimens of irinotecan and oxaliplatin, no significant differences were found in different NRA groups with OS. However, oxaliplatin‐based chemotherapy was markedly associated with decreased DCR in high‐NRA groups. Notably, we demonstrated that oxaliplatin decreased DCR in 3–4 NRA group patients, whereas irinotecan increased DCR as a whole. The inconsistent results may be the discrepancy in the principle of drug action. Irinotecan may result in more gastrointestinal reactions, whereas oxaliplatin is primarily associated with immune responses and neurotoxicity.^48^, ^49^, ^50^, ^51^ Therefore, we supposed that genetic variations could regulate disease progression and susceptibility to drugs by affecting gene expression levels. Hence, we conducted the eQTL analysis from the TCGA database. It revealed that rs10890208 and rs3862218 were eQTL for *YBX1* in colorectal cancer tissue. However, this study shows that the expression of *YBX1* has no significant effect on the OS of patients in the TCGA database. Based on these results, it was presumed that the genetic variants of rs10890208 and rs3862218 may affect the *YBX1* expression level by different functional mechanisms. But due to the limitations of the TCGA database, some patients did not have clear information about receiving concrete therapy, whether an abnormal expression of *YBX1* can affect the survival time of patients receiving chemotherapy and its sensitivity to irinotecan or oxaliplatin needs to be verified utilizing sophisticated data.

However, several limitations are present in this study. On the one hand, we did not use an independent study to validate the association between the candidate SNPs and chemotherapy efficacy of colorectal cancer patients. On the other hand, for the molecular biological functions of rs10890208 and rs3862218, further functional experiments are needed to clarify their specific regulatory mechanisms for *YBX1*.

In recent years, several pieces of evidence have explained the regulatory role of RNA m^5^C modification in posttranscription.^52^ In the study of the pathogenesis and progression of cancer, RNA m^5^C modification genes have been reported to be relevant to several cancers, including bladder cancers, hepatocellular carcinoma, glioblastoma multiforme, and leukemia.^53^ A few studies have reported genetic variations in genes as a predictor of drug resistance in colorectal cancer patients.^54^ In this study, we found that rs10890208 and rs3862218 in *YBX1* were associated with survival time in chemotherapy patients. Thus, we further evaluated the combined effect of SNPs on overall survival and disease control rates by adding the alleles into the number of risk alleles. In summary, this study was the first study that focus on the association between genetic variations of m^5^C modification genes and survival time and drug sensitivity after chemotherapy for colorectal cancer patients. Interestingly, our study found that the two chemotherapy regimens had significantly different effects on DCR in patients with colorectal cancer. These findings, based on previous literature, further suggest the value of genetic variations of *YBX1* in predicting the efficacy of colorectal cancer and provide a new basis for the prediction of early detection markers of chemotherapy resistance.

## AUTHOR CONTRIBUTIONS

SL and QY designed the study and reviewed the manuscript; SC, XC, and SB performed the statistical analysis and wrote the manuscript; XC and SB reviewed and check the final data; LZ and DG contributed technical and material support; and YW provided critical comments.

## FUNDING INFORMATION

This study was supported by the Gusu Health Talents Program (GSWS2021040), the Natural Science Foundation of the Jiangsu Higher Education Institutions of China (20KJB330008), and the Translational Medicine Research Program of the Wuxi Health Care Committee (ZH202102).

## CONFLICT OF INTEREST

The authors declare that they have no conflict of interest.

## ETHICS APPROVAL AND CONSENT TO PARTICIPATE

The study was approved by the institution review board of Nanjing Medical University.

## Supporting information


Figure S1
Figure S2
Figure S3
Figure S4
Figure S5
Figure S6

Table S1
Table S3
Table S4
Table S5
Table S6
Table S7
Table S8

## Data Availability

The data that support the findings of this study are available within the Supporting Information files and from the corresponding author upon reasonable request.
