# Supplementary material for: Genetic variants in RNA m5C modification genes associated with survival and chemotherapy efficacy of colorectal cancer
Source: Cancer Med. 2022 Jul 21;12(2):1376–88. doi: 10.1002/cam4.5018 (PMC9883553; doi:10.1002/cam4.5018)

**Supplementary Figure legends**

**Figure S1. Interaction of key genes in m^5^C modification.** (A) The relationship between 13 key genes in m^5^C modification. (B) Protein-protein interaction (PPI) network among 11 m^5^C modification enzymes. NSUN5P1 and NSUN5P2 were unavailable in STRING.

**Figure S2. Annotation for SNP rs10890208 and rs3862218.** (A) The location of the two SNPs in the genome. Exons for the *YBX1* gene are shown at the top as boxes and introns as the intervening line. (B) The predicted m^5^C sites in *YBX1* on chromosome 1. Enrichment of the histone ChIP-Seq (H3K27ac, H3K36me3, H3K4me1, H3K4me3) in the transverse colon (C) and sigmoid colon tissue (D). (E) Integrative and epigenetics score specific to rs10890208 and rs3862218 in FAVOR.

**Figure S3. RNA secondary structure diagram and the minimum free energy (MFE) for SNP rs10890208 and rs3862218.** (A) rs10890208 wild-type. (B) rs10890208 mutant. (C) rs3862218 wild-type. (D) rs3862218 mutant.

**Figure S4. Correlation analysis of the mRNA expression level between candidate transcription factors and *YBX1* in TCGA.** (A) Correlation analysis of the mRNA expression level between transcription factor *CTCF* and *YBX1* and three different genotypes (rs10890208 CC, rs10890208 CA, rs10890208 AA) in the TCGA database. (B-D) Correlation analysis of the mRNA expression level between transcription factor *POLR2A*, *SMARCA4*, *TRIM28*, and *YBX1* and three different genotypes (rs3862218 AA, rs3862218 AG, rs3862218 GG) in the TCGA database.

**Figure S5. Scatter plot for genetic associations of the two SNPs and** **phenome.** The Open Targets Genetics Portal was used to visualize the genetic associations of rs10890208 (A) and rs3862218 (B) and phenome. Traits with *P*-value < 0.01 was shown in the scatter plot.

**Figure S6. Association between expression of *YBX1* and** **colorectal cancer development in the TCGA database.** (A) Forest plot for *YBX1* expression level and the prognosis of 33 cancer types using Cox regression. Based on online survival analysis software (GEPIA2), the quartile was used as the cut-off value. Each point and horizontal line represent HR and 95% CI. (B) Expression of *YBX1* stratified by tumor stages. (C) Multivariable adjusted logistic regression coefficients for *YBX1* mRNA expression and colorectal cancer stage. ORs are indicated by solid lines and 95% CIs by shaded areas derived from restricted cubic spline regressions with five knots. Adjusted for age and sex. **P* < 0.05; ***P* < 0.01; ****P* < 0.001.

**Figure S1.**


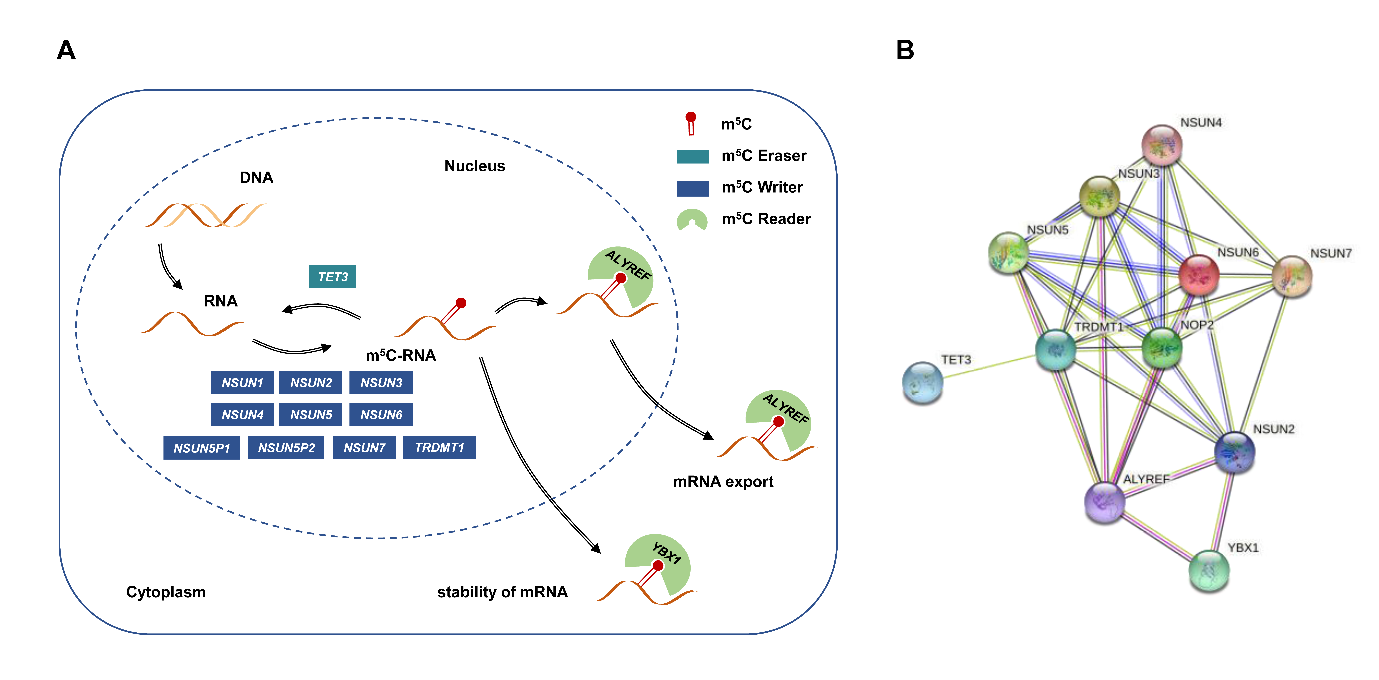


**Figure S2.**


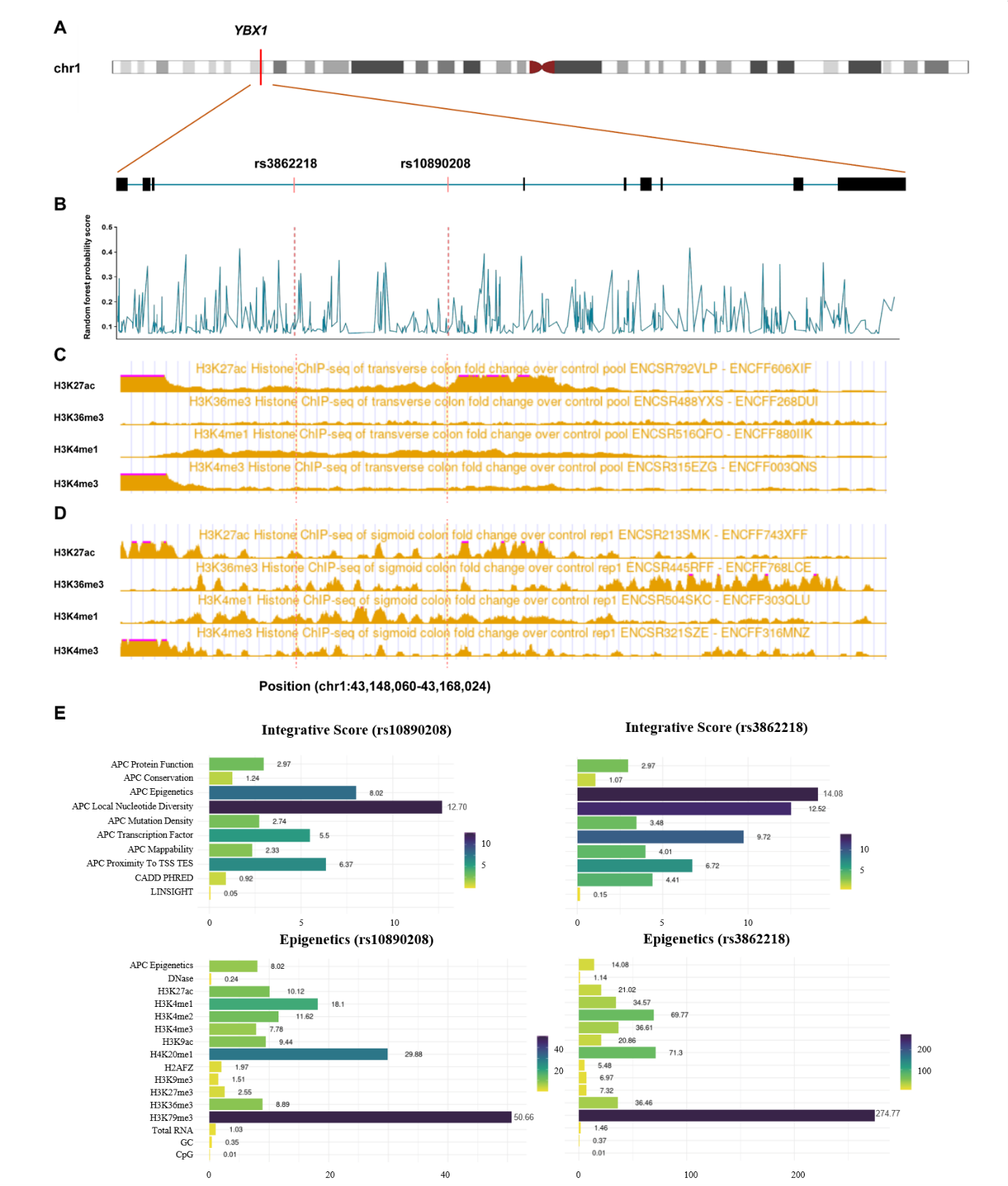


**Figure S3.**


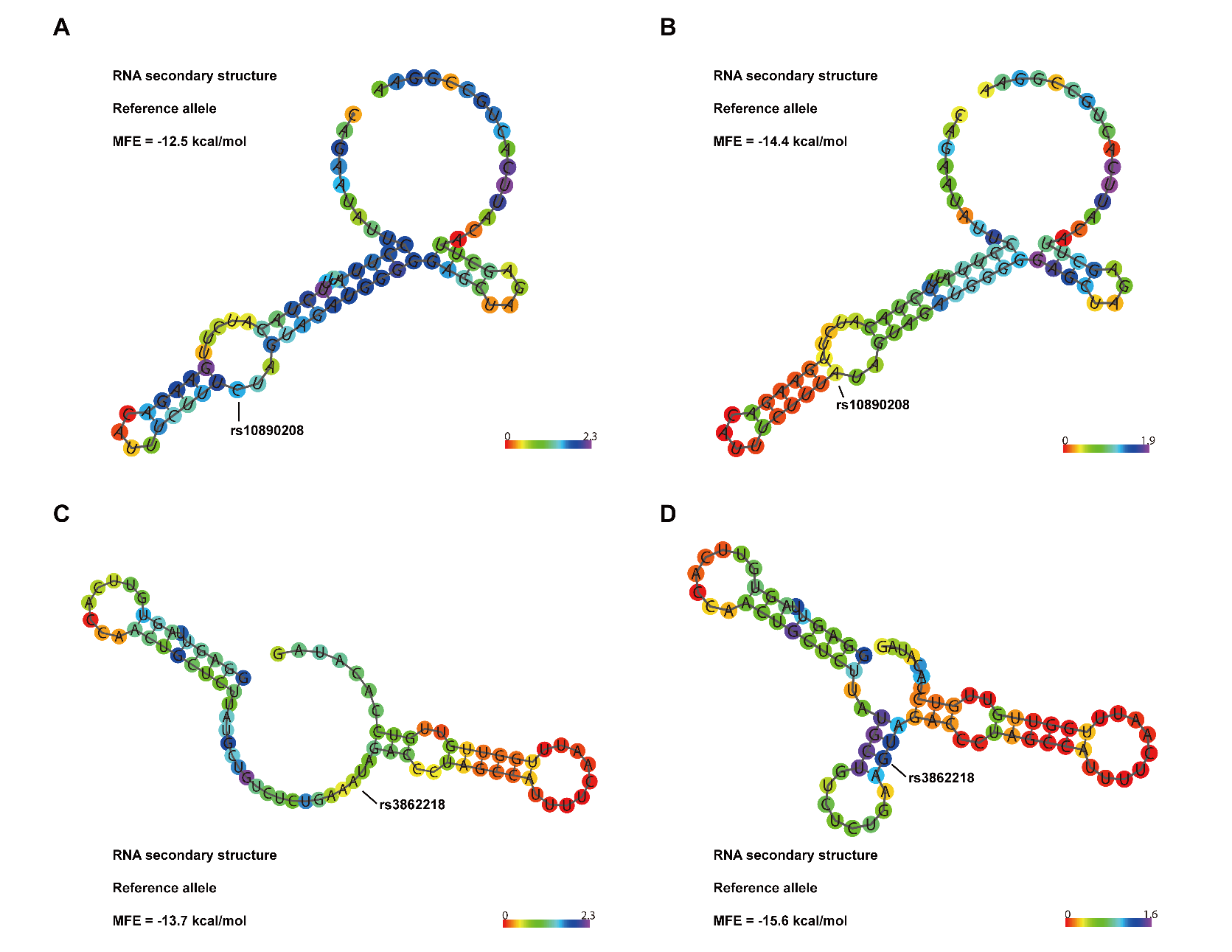


**Figure S4.**


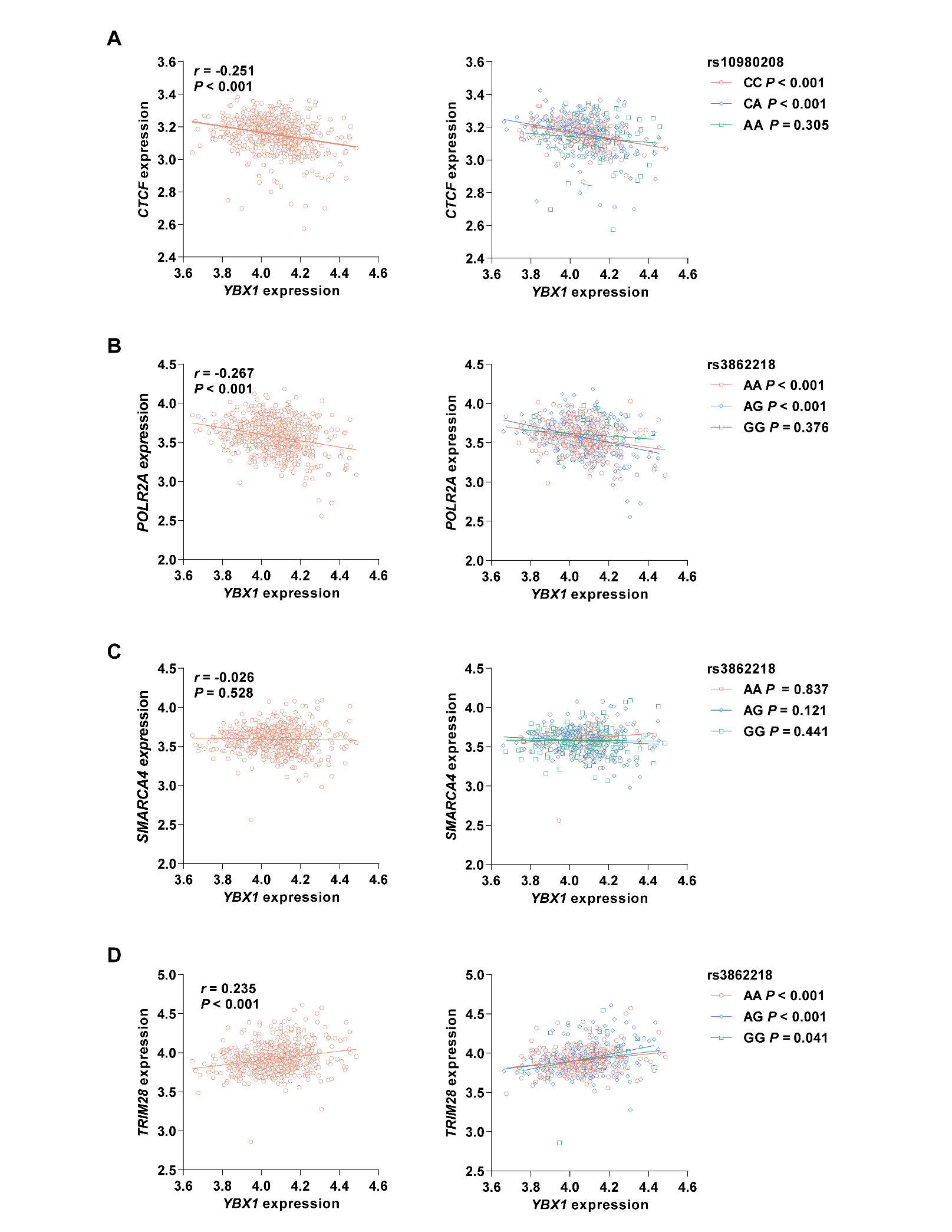


**Figure S5.**


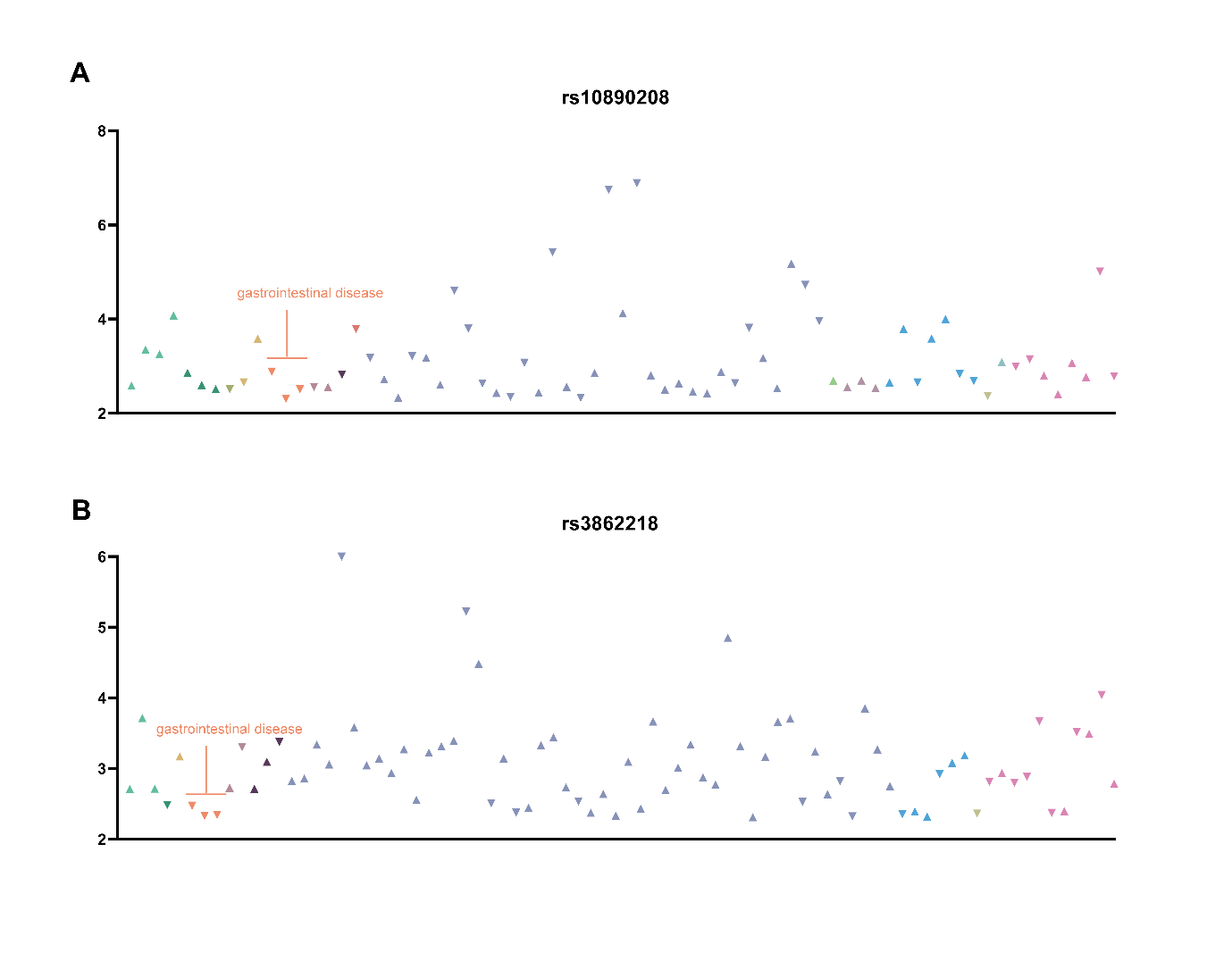


**Figure S6.**


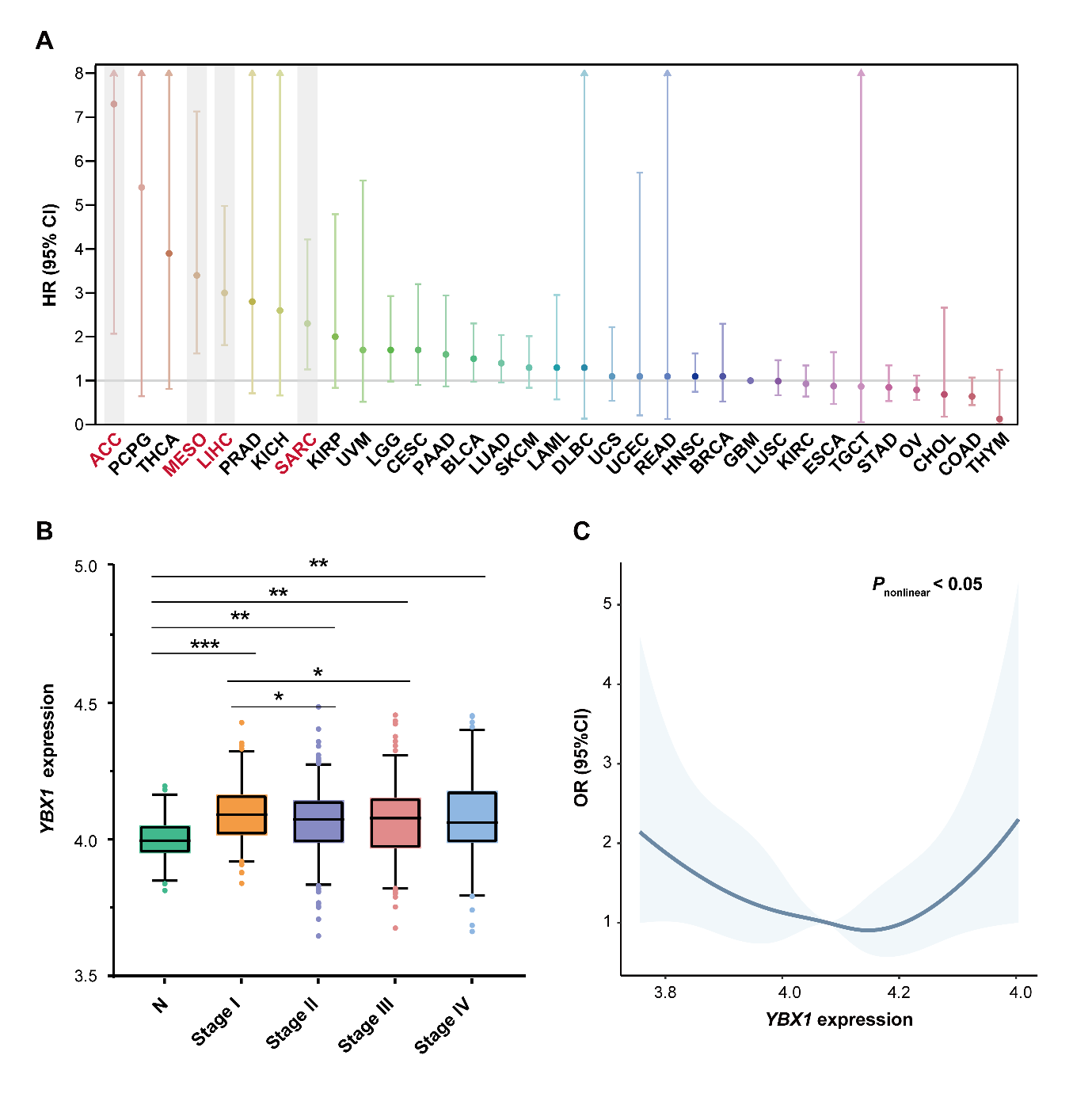

Supplement: Supplementary file 1 — Figure S1 Figure S2 Figure S3 Figure S4 Figure S5 Figure S6 [file CAM4-12-1376-s002.docx]
