# Supplementary material for: Genetic variants in RNA m5C modification genes associated with survival and chemotherapy efficacy of colorectal cancer
Source: Cancer Med. 2022 Jul 21;12(2):1376–88. doi: 10.1002/cam4.5018 (PMC9883553; doi:10.1002/cam4.5018)
Supplement: Supplementary file 2 — Table S1 Table S3 Table S4 Table S5 Table S6 Table S7 Table S8 [file CAM4-12-1376-s001.docx]

**Table S1.** Previously reported studies on m^5^C modification genes.

| **Chr** | **Genes** | **Position(hg19)** | **Roles** | **Related cancer types^a^** | **First author** | **Year** | **PMID** |
| --- | --- | --- | --- | --- | --- | --- | --- |
| 1 | *NSUN4* | 46806479-46850871 | Writer | Breast and prostate cancer | Kar et al. | 2016 | 27432226 |
| 1 | *YBX1* | 43148089-43169476 | Reader | Breast cancer/ Lung cancer/ Colorectal cancer/ Bladder cancer/ Hyroid neoplasms/ Glioblastoma multiforme | Barguo et al. | 1997 | 9095180 |
| 2 | *TET3* | 74212037-74335303 | Eraser | glioblastoma stem cells/ Breast cancer/ Colorectal cancer/ Gastric cancer | Cui et al. | 2016 | 26838672 |
| 3 | *NSUN3* | 93781905-93850676 | Writer | Leukaemia | Cheng et al. | 2018 | 29563491 |
| 4 | *NSUN7* | 40751972-40813201 | Writer | NR | Chi et al. | 2013 | 23816522 |
| 5 | *NSUN2* | 6599352-6633157 | Writer | Squamous cell carcinoma/ Breast cancer/ Leukaemia/ Bladder cancer | Frye et al. | 2006 | 16713953 |
| 7 | *NSUN5* | 72716513-72722823 | Writer | Glioma | Janin et al. | 2019 | 31428936 |
| 7 | *NSUN5P1* | 75039605-75046071 | Writer | NR | Janin et al. | 2019 | 31428936 |
| 7 | *NSUN5P2* | 72418832-72425302 | Writer | NR | Janin et al. | 2019 | 31428936 |
| 10 | *NSUN6* | 18834490-18948167 | Writer | Breast cancer | Li et al. | 2017 | 28114269 |
| 10 | *TRDMT1* | 17179335-17243671 | Writer | Hepatocarcinogenesis/ prostate cancer/ Colorectal cancer/ Somatic cancer/ Gastric cancer | Pang et al. | 2003 | 11230735 |
| 12 | *NSUN1* | 6666037-6677457 | Writer | Breast cancer/ Lung cancer/ Leukaemia/ Colorectal cancer/ prostate adenocarcinoma/ oral carcinoma | Freeman et al. | 1991 | 1672621 |
| 17 | *ALYREF* | 79845711-79849463 | Reader | oral squamous cell carcinoma/ Bladder Cancer/ Breast cancer | Domínguez-Sánchez et al. | 2011 | 21329510 |

NR, the roles of genes in which cancer types were not reported.

^a^Pertinent literature on m^5^C modifications in different cancers has been searched for all relevant reports updated to Dec 1, 2021.

**Table S2.** Functional annotation for selected 63 SNPs.

| **Variant** | **Genes** | **TFBS^a^** | **Regulome DB score^b^** | **Promoter histone marks^c^** | **Enhancer histone marks^c^** | **DNAse^c^** | **Proteins bound^c^** | **Motifs changed^c^** | **GRASP QTL hits^c^** | **Selected eQTL hits^c^** | **Location** |
| --- | --- | --- | --- | --- | --- | --- | --- | --- | --- | --- | --- |
| rs3862218 | *YBX1* | -- | 1d | 7 tissues | 16 tissues | 5 tissues | -- | *Irf, TATA, YY1* | 17 hits | 36 hits | Intronic |
| rs10890208 | *YBX1* | -- | 4 | -- | -- | -- | *CTCF* | *GR* | -- | 12 hits | Intronic |
| rs10493113 | *YBX1* | -- | 1f | -- | -- | 5 tissues | *--* | 4 altered motifs | 4 hits | 8 hits | Intronic |
| rs12030724 | *YBX1* | -- | 5 | -- | BLD, GI, PANC | -- | *--* | *Bbx, DMRT2, Hbp1* | -- | 2 hits | Intronic |
| rs11590411 | *NSUN4* | Y | 5 | -- | BLD, CRVX | -- | *--* | 7 altered motifs | -- | 25 hits | -- |
| rs10736428 | *NSUN4* | Y | 5 | -- | -- | BLD | *--* | 13 altered motifs | -- | 4 hits | -- |
| rs7555989 | *NSUN4* | -- | 3a | -- | SKIN, GI, BLD |  | *--* | -- | -- | 3 hits | Intronic |
| rs56737734 | *NSUN4* | -- | 7 | -- | -- | -- | *--* | 4 altered motifs | -- | 22 hits | Intronic |
| rs10207365 | *TET3* | -- | 6 | -- | -- | -- | *--* | 4 altered motifs | -- | -- | -- |
| rs828872 | *TET3* | -- | 7 | -- | PLCNT, HRT | -- | *--* | *INSM1, RXRA* | -- | -- | Intronic |
| rs7589197 | *TET3* | -- | 4 | -- | BRN, GI | -- | *--* | *SETDB1, ZID* | -- | -- | Intronic |
| rs11903339 | *TET3* | -- | 6 | -- | -- | -- | *--* | *Hoxa10, Hoxd10* | -- | -- | Intronic |
| rs828867 | *TET3* | -- | 4 | ESC, ESDR, IPSC | 4 tissues | 4 tissues | *POL24H8, POL2, ZNF263* |  | -- | -- | 3'-UTR |
| rs6546891 | *TET3* | -- | 4 | ESC, IPSC | ESC, ESDR, IPSC | -- | *--* | 4 altered motifs | 1 hit | 56 hits | 3'-UTR |
| rs828866 | *TET3* | -- | 4 | -- | -- | -- | -- | *Nrf1, Pbx-1, Rad21* | -- | 51 hits | -- |
| rs17855824 | *NSUN3* | -- | 5 | -- | BLD | -- | -- | 4 altered motifs | -- | -- | Missense |
| rs9843295 | *NSUN3* | -- | 5 | BLD | BLD | -- | -- | *NF-E2* | -- | 4 hits | Intronic |
| rs10934676 | *NSUN3* | -- | 4 | -- | -- | -- | -- | *SP2* | 2 hits | 15 hits | Intronic |
| rs2965 | *NSUN3* | -- | 1f | -- | ESDR, BLD, ADRL | ESDR | *GATA2* | *Foxa, Ik-3, STAT* | 1 hit | 2 hits | Intronic |
| rs6809577 | *NSUN3* | -- | 7 | -- | -- | -- | -- | 5 altered motifs | -- | 4 hits | Intronic |
| rs921727 | *NSUN3* | -- | 4 | -- | SKIN | -- | -- | *DMRT3, DMRT7* | -- | 1 hit | Intronic |
| rs9860972 | *NSUN3* | -- | 7 | -- | -- | -- | -- | *AIRE* | -- | 6 hits | Intronic |
| rs55690540 | *NSUN7* | Y | 6 | -- | -- | -- | -- | 5 altered motifs | -- | -- | -- |
| rs2437333 | *NSUN7* | -- | 4 | 8 tissues | ESDR, BRST, HRT | -- | -- | 5 altered motifs | -- | 10 hits | Intronic |
| rs56858776 | *NSUN7* | -- | 5 | -- | -- | -- | -- | 4 altered motifs | -- | -- | Intronic |
| rs6834203 | *NSUN7* | -- | 1f | -- | -- | -- | -- | *GR, Irx* | 1 hit | 4 hits | Intronic |
| rs11724316 | *NSUN7* | Y | 7 | -- | -- | -- | -- | *AP-2rep* | -- | 1 hit | Intronic |
| rs2437314 | *NSUN7* | Y | 7 | -- | -- | -- | -- | *LBP-1, LBP-9* | 1 hit | 1 hit | Intronic |
| rs4861312 | *NSUN7* | -- | 6 | -- | -- | -- | -- | *CDP, HNF4* | -- | -- | Intronic |
| rs7663370 | *NSUN7* | -- | 5 | -- | BLD, LIV | -- | -- | *Mrg1, Hoxa9, YY1* | -- | 1 hit | Intronic |
| rs56918153 | *NSUN7* | -- | 5 | BLD | BLD, LIV | BLD, BLD | -- | 8 altered motifs | -- | -- | Intronic |
| rs62303771 | *NSUN7* | -- | 5 | BLD | BLD, LIV | -- | -- | *Pax-5* | -- | 13 hits | Intronic |
| rs545493 | *NSUN2* | -- | 1f | -- | 4 tissues | -- | -- | *SRF, THAP1, YY1* | 6 hits | 28 hits | -- |
| rs11743743 | *NSUN2* | -- | 2b | -- | 8 tissues | 5 tissues | *POL2S2, ZNF274* | 8 altered motifs | -- | 8 hits | -- |
| rs6876835 | *NSUN2* | -- | 4 | -- | LIV | -- | *--* | 4 altered motifs | 3 hits | 3 hits | -- |
| rs3822434 | *NSUN2* | -- | 3a | -- | LIV | -- | *POL2, POL24H8* |  | -- | 5 hits | Missense |
| rs3776444 | *NSUN2* | -- | 1f | -- | -- | -- | *POL2, POL24H8* | 8 altered motifs | 6 hits | 47 hits | Intronic |
| rs3776443 | *NSUN2* | -- | 4 | -- | -- | -- | *POL2, POL24H8* | *YY1* | -- | 8 hits | Intronic |
| rs4701741 | *NSUN2* | -- | 1f | -- | -- | -- | -- | 7 altered motifs | 3 hits | 26 hits | Intronic |
| rs6555404 | *NSUN2* | -- | 5 | -- | SKIN | -- | -- | *BCL, HNF4* | -- | 10 hits | Intronic |
| rs484418 | *NSUN2* | -- | 1d | -- | SKIN | -- | -- | *HES1, PEBP, SEF-1* | 4 hits | 29 hits | Intronic |
| rs3776437 | *NSUN2* | -- | 1f | -- | STRM, SKIN | -- | -- | -- | 1 hit | 20 hits | Intronic |
| rs529439 | *NSUN2* | -- | 7 | -- | MUS, SKIN | -- | -- | -- | -- | 1 hit | Intronic |
| rs16877698 | *NSUN2* | -- | 6 | -- | -- | -- | -- | 8 altered motifs | 1 hit | 12 hits | Intronic |
| rs11744948 | *NSUN2* | -- | 6 | -- | -- | -- | -- | *Mef2, ZBTB33* |  | 12 hits | Intronic |
| rs518673 | *NSUN2* | Y | 4 | -- | BRST, BLD, LIV |  | -- | -- | 1 hit | 1 hit | Intronic |
| rs166049 | *NSUN2* | Y | 3a | 23 tissues | 5 tissues | LNG, CRVX | -- | 6 altered motifs |  | 24 hits | Intronic |
| rs8192120 | *NSUN2* | Y | 4 | 23 tissues | 5 tissues | CRVX | -- | -- | 1 hit | 3 hits | Intronic |
| rs531290 | *NSUN2* | Y | 4 | 20 tissues | 9 tissues | 16 tissues | *PU1* | 4 altered motifs | -- | 7 hits | Intronic |
| rs1880948 | *NSUN5* | Y | 4 | 24 tissues | BLD, BRN, GI | 38 tissues | 30 bound proteins |  | -- | 4 hits | -- |
| rs12355391 | *TRDMT1* | -- | 5 | -- | -- | -- | -- | 14 altered motifs | -- | 9 hits | Intronic |
| rs10795453 | *TRDMT1* | -- | 3a | -- | -- | -- | -- | *AP-4, Crx, Pitx2* | -- | -- | Intronic |
| rs11254419 | *TRDMT1* | -- | 5 | -- | GI | -- | -- | 5 altered motifs | -- | 24 hits | Intronic |
| rs10795454 | *TRDMT1* | -- | 7 | -- | -- | -- | -- | 6 altered motifs | -- | 22 hits | Intronic |
| rs10904904 | *TRDMT1* | -- | 4 | -- | -- | BLD, KID, BLD | *CEBPB* | 10 altered motifs | -- | 19 hits | Intronic |
| rs7082679 | *TRDMT1* | Y | 4 | 24 tissues | -- | 49 tissues | *CTCF* | *ATF3* | -- | 2 hits | Intronic |
| rs3765181 | *NSUN6* | -- | 5 | -- | -- | HRT | -- | 7 altered motifs | -- | -- | Intronic |
| rs11525266 | *NSUN6* | -- | 6 | -- | -- | -- | -- | *Hoxa9, Hoxb9* | -- | 44 hits | Intronic |
| rs7081972 | *NSUN6* | -- | 7 | -- | 12 tissues | -- | -- | *BCL* | -- | 18 hits | Intronic |
| rs4468242 | *NSUN6* | -- | 7 | -- | LIV | -- | -- | 4 altered motifs | 1 hit | 6 hits | Intronic |
| rs2267967 | *NSUN1* | -- | 4 | -- | -- | -- | -- | *Pou3f2, SRF, p300* | -- | -- | Intronic |
| rs1558333 | *NSUN1* | -- | 5 |  | BLD | -- | -- | Smad3, Smad | 15 hits | 15 hits | Intronic |
| rs3764909 | *NSUN1* | Y | 4 | 24 tissues | -- | 38 tissues | 4 bound proteins | *AhR, NRSF, Sin3Ak-20* | -- | -- | Intronic |

^a^TFBS: Transcription factors binding sites, based on SNPinfo Web Server (http://snpinfo.niehs.nih.gov/).

^b^Regulome DB score: 1d, eQTL + TF binding + any motif + DNase peak; 1f, eQTL + TF binding / DNase peak; 4, TF binding + DNase peak; 6, other, based on Regulome DB (http://regulome.stanford.edu/).

^c^Based on HaploReg v4.1 (http://compbio.mit.edu/HaploReg).

**Table S3.** The association of 46 SNPs with OS of colorectal cancer patients treated with chemotherapy.

| **Variant** | **Position (hg19)** | **Allele^a^** | **MAF^b^** | **Gene** | **OS** | | |
| --- | --- | --- | --- | --- | --- | --- | --- |
|  |  |  |  |  | **HR (95% CI)^c^** | ***P*^c^** | **FPRP** |
| rs3862218 | 1:43152610 | A/G | 0.165 | *YBX1* | 1.36 (1.04-1.78) | 0.025 | 0.184 |
| rs10890208 | 1:43156542 | C/A | 0.355 | *YBX1* | 1.43 (1.15-1.78) | 0.001 | 0.019 |
| rs10493113 | 1:43158510 | C/T | 0.188 | *YBX1* | 1.25 (0.96-1.65) | 0.101 | 0.501 |
| rs12030724 | 1:43160018 | A/T | 0.256 | *YBX1* | 0.73 (0.56-0.97) | 0.028 | 0.251 |
| rs11590411 | 1:46804254 | G/T | 0.164 | *NSUN4* | 1.08 (0.79-1.48) | 0.634 | 0.853 |
| rs10736428 | 1:46804402 | A/C | 0.132 | *NSUN4* | 1.13 (0.80-1.61) | 0.477 | 0.820 |
| rs7555989 | 1:46813121 | C/A | 0.477 | *NSUN4* | 1.04 (0.81-1.33) | 0.778 | 0.875 |
| rs7589197 | 2:74322469 | T/C | 0.360 | *TET3* | 1.01 (0.79-1.30) | 0.918 | 0.892 |
| rs828867 | 2:74334462 | G/A | 0.463 | *TET3* | 1.12 (0.89-1.41) | 0.340 | 0.755 |
| rs6546891 | 2:74334790 | A/G | 0.491 | *TET3* | 1.03 (0.82-1.29) | 0.796 | 0.878 |
| rs828866 | 2:74336274 | T/A | 0.117 | *TET3* | 0.88 (0.61-1.27) | 0.497 | 0.827 |
| rs17855824 | 3:93779860 | C/T | 0.065 | *NSUN3* | 1.12 (0.69-1.80) | 0.644 | 0.867 |
| rs9843295 | 3:93790542 | A/G | 0.218 | *NSUN3* | 0.85 (0.63-1.13) | 0.260 | 0.712 |
| rs10934676 | 3:93813171 | C/G | 0.129 | *NSUN3* | 0.92 (0.67-1.28) | 0.627 | 0.853 |
| rs2965 | 3:93818466 | T/G | 0.440 | *NSUN3* | 0.92 (0.73-1.15) | 0.450 | 0.802 |
| rs921727 | 3:93833508 | A/C | 0.457 | *NSUN3* | 0.93 (0.73-1.17) | 0.537 | 0.829 |
| rs2437333 | 4:40753522 | A/G | 0.458 | *NSUN7* | 1.01 (0.80-1.28) | 0.920 | 0.892 |
| rs56858776 | 4:40772549 | G/C | 0.346 | *NSUN7* | 0.93 (0.72-1.19) | 0.546 | 0.832 |
| rs6834203 | 4:40780364 | T/C | 0.394 | *NSUN7* | 0.90 (0.71-1.14) | 0.397 | 0.782 |
| rs7663370 | 4:40808779 | C/A | 0.499 | *NSUN7* | 1.05 (0.83-1.33) | 0.685 | 0.861 |
| rs56918153 | 4:40809645 | G/T | 0.270 | *NSUN7* | 0.91 (0.70-1.18) | 0.465 | 0.809 |
| rs62303771 | 4:40809796 | C/A | 0.472 | *NSUN7* | 0.97 (0.76-1.22) | 0.783 | 0.876 |
| rs545493 | 5:6597622 | T/C | 0.149 | *NSUN2* | 0.95 (0.69-1.32) | 0.773 | 0.876 |
| rs11743743 | 5:6598553 | C/A | 0.455 | *NSUN2* | 0.98 (0.78-1.23) | 0.831 | 0.882 |
| rs6876835 | 5:6599222 | G/A | 0.359 | *NSUN2* | 1.04 (0.82-1.31) | 0.765 | 0.873 |
| rs3822434 | 5:6600150 | G/A | 0.242 | *NSUN2* | 0.94 (0.71-1.23) | 0.642 | 0.853 |
| rs3776444 | 5:6601055 | T/A | 0.370 | *NSUN2* | 0.93 (0.74-1.17) | 0.526 | 0.826 |
| rs3776443 | 5:6601066 | C/T | 0.422 | *NSUN2* | 1.05 (0.84-1.31) | 0.664 | 0.857 |
| rs4701741 | 5:6601309 | T/C | 0.146 | *NSUN2* | 0.95 (0.68-1.32) | 0.747 | 0.873 |
| rs6555404 | 5:6606445 | G/A | 0.209 | *NSUN2* | 1.03 (0.79-1.34) | 0.836 | 0.883 |
| rs484418 | 5:6608877 | G/A | 0.118 | *NSUN2* | 1.09 (0.78-1.54) | 0.612 | 0.851 |
| rs3776437 | 5:6609088 | C/A | 0.414 | *NSUN2* | 0.95 (0.75-1.21) | 0.672 | 0.858 |
| rs518673 | 5:6629930 | G/A | 0.257 | *NSUN2* | 0.90 (0.69-1.19) | 0.467 | 0.810 |
| rs166049 | 5:6632295 | T/G | 0.232 | *NSUN2* | 0.91 (0.68-1.20) | 0.498 | 0.820 |
| rs8192120 | 5:6632320 | A/C | 0.260 | *NSUN2* | 1.03 (0.80-1.33) | 0.813 | 0.880 |
| rs531290 | 5:6634688 | C/G | 0.373 | *NSUN2* | 0.94 (0.74-1.20) | 0.613 | 0.847 |
| rs1880948 | 7:72722981 | A/G | 0.359 | *NSUN5* | 0.82 (0.63-1.06) | 0.127 | 0.549 |
| rs12355391 | 10:17201546 | A/C | 0.370 | *TRDMT1* | 0.91 (0.71-1.18) | 0.489 | 0.816 |
| rs10795453 | 10:17208638 | A/G | 0.357 | *TRDMT1* | 0.89 (0.70-1.13) | 0.349 | 0.760 |
| rs11254419 | 10:17209192 | C/A | 0.274 | *TRDMT1* | 1.30 (1.00-1.69) | 0.047 | 0.329 |
| rs10904904 | 10:17240640 | G/C | 0.427 | *TRDMT1* | 0.90 (0.71-1.15) | 0.415 | 0.790 |
| rs7082679 | 10:17243046 | T/G | 0.322 | *TRDMT1* | 0.93 (0.72-1.18) | 0.537 | 0.829 |
| rs3765181 | 10:18836931 | G/A | 0.385 | *NSUN6* | 1.00 (0.80-1.24) | 0.970 | 0.897 |
| rs2267967 | 12:6671799 | C/T | 0.279 | *NSUN1* | 1.06 (0.83-1.35) | 0.641 | 0.853 |
| rs1558333 | 12:6675055 | T/G | 0.340 | *NSUN1* | 1.00 (0.73-1.36) | 0.977 | 0.898 |
| rs3764909 | 12:6677197 | C/A | 0.342 | *NSUN1* | 1.02 (0.80-1.30) | 0.885 | 0.889 |

OS, overall survival; MAF, minor allele frequency; HR, hazard ratio; CI, confidence interval; FPRP, false-positive report probability.

^a^Major allele/minor allele.

^b^Minor allele frequencies were calculated using the in-house data.

^c^Adjusted for age, sex, smoking, and drinking status in the additive genetic model in Cox regression models.

**Table S4.** Stratified analysis of the associations between rs10890208 and rs3862218 and OS after chemotherapy in the dominant model.

| **Variables** | **rs10890208** | | |  | **rs3862218** | | |
| --- | --- | --- | --- | --- | --- | --- | --- |
|  | **HR (95% CI)^a^** | ***P*^a^** | ***P*_interaction_** |  | **HR (95% CI)^a^** | ***P*^a^** | ***P*_interaction_** |
| Age (years) |  |  |  |  |  |  |  |
| ≤60 | 1.89 (1.18-3.02) | 0.008 | 0.661 |  | 1.25 (0.74-2.11) | 0.397 | 0.317 |
| >60 | 1.51 (0.92-2.48) | 0.105 |  |  | 1.59 (0.97-2.59) | 0.064 |  |
| Sex |  |  |  |  |  |  |  |
| Female | 1.70 (0.91-3.14) | 0.094 | 0.554 |  | 2.08 (1.16-3.75) | 0.015 | 0.114 |
| Male | 1.57 (1.06-2.33) | 0.026 |  |  | 1.23 (0.80-1.90) | 0.342 |  |
| Smoking status |  |  |  |  |  |  |  |
| Never | 1.51 (0.87-2.62) | 0.142 | 0.650 |  | 1.23 (0.67-2.27) | 0.499 | 0.798 |
| Ever | 1.70 (1.10-2.63) | 0.016 |  |  | 1.52 (0.98-2.34) | 0.059 |  |
| Drinking status |  |  |  |  |  |  |  |
| Never | 2.28 (1.16-4.50) | 0.017 | 0.533 |  | 1.56 (0.83-2.94) | 0.168 | 0.943 |
| Ever | 1.54 (1.04-2.29) | 0.031 |  |  | 1.46 (0.96-2.23) | 0.078 |  |
| Tumor site |  |  |  |  |  |  |  |
| Colon | 1.36 (0.87-2.12) | 0.176 |  |  | 1.24 (0.76-2.04) | 0.386 |  |
| Rectum | 2.39 (1.40-4.05) | 0.001 |  |  | 1.86 (1.10-3.12) | 0.020 |  |
| Tumor grade |  |  |  |  |  |  |  |
| Well+Moderate | 1.51 (1.04-2.21) | 0.032 |  |  | 1.39 (0.92-2.10) | 0.116 |  |
| Poor | 1.98 (0.92-4.30) | 0.082 |  |  | 1.43 (0.70-2.90) | 0.323 |  |
| Dukes stage |  |  |  |  |  |  |  |
| C | 0.12 (0.01-1.85) | 0.127 |  |  | 0.07 (0.00-2.13) | 0.125 |  |
| D | 1.76 (1.25-2.47) | 0.001 |  |  | 1.54 (1.08-2.19) | 0.017 |  |
| Metastasis^b^ |  |  |  |  |  |  |  |
| ≤2 | 1.51 (1.03-2.22) | 0.033 |  |  | 1.44 (0.96-2.14) | 0.074 |  |
| >2 | 2.44 (1.03-5.78) | 0.043 |  |  | 1.91 (0.83-4.41) | 0.131 |  |
| Treatment |  |  |  |  |  |  |  |
| Oxaliplatin | 1.54 (0.99-2.40) | 0.056 |  |  | 1.72 (1.06-2.80) | 0.029 |  |
| Irinotecan | 1.75 (1.03-2.96) | 0.038 |  |  | 1.19 (0.71-1.98) | 0.505 |  |

OS, overall survival; HR, hazard ratio; CI, confidence interval.

^a^Adjusted for age, sex, smoking, and drinking status in Cox regression models; Some cases were not included due to incomplete overall survival information.

^b^Some cases were not included due to missing clinical data.

**Table S5.** Association of rs10890208 and rs3862218 with PFS after chemotherapy and DCR of colorectal cancer in four genetic models.

| **Models** | **PFS** | |  | **DCR** | |
| --- | --- | --- | --- | --- | --- |
|  | **HR (95% CI)^a^** | ***P^a^*** |  | **OR (95% CI)^b^** | ***P*^b^** |
| rs10890208 |  |  |  |  |  |
| CC | 1.00 |  |  | 1.00 |  |
| CA | 1.01 (0.76-1.34) | 0.941 |  | 1.01 (0.55-1.85) | 0.976 |
| AA | 1.05 (0.71-1.56) | 0.789 |  | 1.08 (0.47-2.35) | 0.855 |
| Additive model | 1.02 (0.85-1.23) | 0.808 |  | 1.03 (0.70-1.51) | 0.872 |
| Dominant model | 1.02 (0.78-1.33) | 0.871 |  | 1.03 (0.59-1.81) | 0.921 |
| Recessive model | 1.05 (0.73-1.51) | 0.796 |  | 1.07 (0.49-2.20) | 0.854 |
| rs3862218 |  |  |  |  |  |
| AA | 1.00 |  |  | 1.00 |  |
| AG | 1.20 (0.89-1.62) | 0.243 |  | 1.15 (0.59-2.17) | 0.674 |
| GG | 1.08 (0.59-2.00) | 0.798 |  | 1.16 (0.25-4.01) | 0.832 |
| Additive model | 1.11 (0.89-1.39) | 0.345 |  | 1.11 (0.67-1.80) | 0.671 |
| Dominant model | 1.18 (0.89-1.56) | 0.259 |  | 1.15 (0.62-2.10) | 0.653 |
| Recessive model | 1.03 (0.56-1.90) | 0.916 |  | 1.11 (0.24-3.81) | 0.873 |
| Number of the risk allele (NRA) |  |  |  |  |  |
| 0 | 1.00 |  |  |  |  |
| 1 | 0.92 (0.65-1.31) | 0.641 |  | 0.98 (0.47-2.01) | 0.963 |
| 2 | 1.07 (0.77-1.48) | 0.693 |  | 0.98 (0.47-1.96) | 0.947 |
| 3 | 1.26 (0.71-2.25) | 0.430 |  | 1.37 (0.40-4.07) | 0.590 |
| 4 | 1.05 (0.56-1.96) | 0.888 |  | 1.13 (0.24-4.03) | 0.860 |
| Trend test |  | 0.538 |  |  | 0.759 |
| 0-2 | 1.00 |  |  | 1.00 |  |
| 3-4 | 1.16 (0.76-1.77) | 0.488 |  | 1.28 (0.51-2.96) | 0.581 |

PFS, progression‐free survival; HR, hazard ratio; CI, confidence interval; DCR, disease control rate; OR, odds ratio; PD, progressive disease; NRA, number of the risk allele. Some cases were not included due to missing clinical data or genotyping.

^a^Adjusted for age, sex, smoking, and drinking status in the Cox regression model.

^b^Adjusted for age, sex, smoking, and drinking status in the logistic regression model.

**Table S6.** Stratified analysis of the associations between NRA and PFS after chemotherapy and DCR in the dominant model.

| **Variables** | **PFS** | |  | **DCR** | | |
| --- | --- | --- | --- | --- | --- | --- |
|  | **HR (95% CI)^a^** | ***P*^a^** |  | **OR (95% CI)^b^** | ***P*^b^** |  |
| Age (years) |  |  |  |  |  |  |
| ≤60 | 1.77 (1.00-3.13) | 0.051 |  | 1.79 (0.52-5.37) | 0.319 |  |
| >60 | 0.78 (0.40-1.50) | 0.448 |  | 0.77 (0.16-2.76) | 0.712 |  |
| Sex |  |  |  |  |  |  |
| Female | 1.81 (0.87-3.74) | 0.110 |  | 2.49 (0.60-9.09) | 0.177 |  |
| Male | 0.94 (0.55-1.59) | 0.806 |  | 0.84 (0.22-2.56) | 0.777 |  |
| Smoking status |  |  |  |  |  |  |
| Never | 0.99 (0.51-1.91) | 0.974 |  | 0.79 (0.16-3.09) | 0.744 |  |
| Ever | 1.39 (0.77-2.48) | 0.274 |  | 1.57 (0.46-4.64) | 0.432 |  |
| Drinking status |  |  |  |  |  |  |
| Never | 1.13 (0.60-2.14) | 0.710 |  | 1.19 (0.29-4.21) | 0.795 |  |
| Ever | 1.33 (0.73-2.44) | 0.353 |  | 1.31 (0.35-4.08) | 0.661 |  |
| Tumor site |  |  |  |  |  |  |
| Colon | 1.03 (0.56-1.87) | 0.930 |  | 1.35 (0.35-4.38) | 0.632 |  |
| Rectum | 1.33 (0.71-2.48) | 0.371 |  | 1.09 (0.27-3.65) | 0.898 |  |
| Tumor grade |  |  |  |  |  |  |
| Well + Moderate | 1.35 (0.84-2.17) | 0.211 |  | 1.65 (0.59-4.25) | 0.318 |  |
| Poor | 0.64 (0.23-1.82) | 0.406 |  | 0.43 (0.02-2.88) | 0.459 |  |
| Dukes stage |  |  |  |  |  |  |
| C^c^ | NA | NA |  | NA | NA |  |
| D | 1.12 (0.73-1.72) | 0.607 |  | 1.13 (0.42-2.71) | 0.789 |  |
| Metastasis^c^ |  |  |  |  |  |  |
| ≤2 | 1.35 (0.86-2.13) | 0.191 |  | 1.45 (0.52-3.69) | 0.446 |  |
| >2 | 0.66 (0.19-2.32) | 0.520 |  | 0.91 (0.04-8.41) | 0.939 |  |
| Treatment |  |  |  |  |  |  |
| Oxaliplatin | 1.03 (0.55-1.93) | 0.924 |  | 1.49 (1.02-2.19) | 0.036 |  |
| Irinotecan | 1.21 (0.67-2.17) | 0.532 |  | 0.89 (0.25-2.78) | 0.852 |  |

NRA, number of the risk allele; PFS, progression‐free survival; HR, hazard ratio; CI, confidence interval; DCR, disease control rate; OR, odds ratio; PD, progressive disease. Some cases were not included due to missing clinical data or genotyping.

^a^Adjusted for age, sex, smoking, and drinking status in the Cox regression model.

^b^The result cannot be calculated owing to the sample size.

^c^Adjusted for age, sex, smoking, and drinking status in the logistic regression model.

**Table S7.** Functional annotation for rs10890208, rs3862218 and SNPs related to them (*r*^2^ > 0.8).

| **Variant** | **Genes** | **Regulome DB score^a^** | **Promoter histone marks^b^** | **Enhancer histone marks^b^** | **DNAse^b^** | **Proteins bound^b^** | **Motifs changed^b^** | **GRASP QTL hits^a^** | **Selected eQTL hits^a^** |
| --- | --- | --- | --- | --- | --- | --- | --- | --- | --- |
| **rs10890208** | *YBX1* (intronic) | 4 | -- | -- | -- | *CTCF* | *GR* | -- | 12 hits |
| rs10493112 | *YBX1* (intronic) | 4 | -- | -- | 4 tissues | *--* | *GATA, Myc* | -- | 15 hits |
| rs11210698 | Upstream of *YBX1* | 4 | -- | SKIN, BRN | ESC | *POL2, POL24H8* | -- | 1 hit | 14 hits |
| rs12044179 | *YBX1* (intronic) | 6 | -- | -- | -- | *--* | -- | 2 hits | 16 hits |
| rs10789424 | *YBX1* (intronic) | 4 | -- | -- | 5 tissues | *--* | 4 altered motifs | -- | 12 hits |
| **rs3862218** | *YBX1* (intronic) | 1d | 7 tissues | 16 tissues | 5 tissues | *--* | *Irf, TATA, YY1* | 17 hits | 36 hits |
| rs6670466 | *YBX1* (intronic) | 1f | 15 tissues | 8 tissues | 11 tissues | *POL2, POL24H8, YY1* | *--* | 4 hits | 37 hits |
| rs11210696 | *YBX1* (intronic) | 1f | -- | 16 tissues | BLD, SKIN | *--* | *Hoxa5, Pou2f2, Pou3f2* | 13 hits | 39 hits |
| rs3895305 | *YBX1* (intronic) | 1f | BLD | 18 tissues | BLD, SKIN | *--* | *CDP, GATA, Ncx* | 11 hits | 36 hits |

^a^Regulome DB score: 1d, eQTL + TF binding + any motif + DNase peak; 1f, eQTL + TF binding / DNase peak; 4, TF binding + DNase peak; 6, other, based on Regulome DB (http://regulome.stanford.edu/).

^b^Based on HaploReg v4.1 (http://compbio.mit.edu/HaploReg).

**Table S8.** Associations between *YBX1* mRNA expression and tumor stage.

|  | **OR (95% CI)** | ***P*-value** |
| --- | --- | --- |
| Model 1^a^ |  |  |
| *YBX1*^c^ | 0.61 (0.19-1.67) | 0.406 |
| Model 2^b^ |  |  |
| *YBX1*^c^ | 0.64 (0.19-2.11) | 0.465 |

^a^Model 1: unadjusted logistic regression model.

^b^Model 2: adjusted for age and sex in the logistic regression model.

^c^*YBX1* mRNA expression in the TCGA database was log10-transformed to fit the regression model (Tumor stage III & IV vs Tumor stage I & II).
